# Navigating the diagnostic challenges of myoclonus in neurodegenerative disorders: video-EEG/polygraphy, clinical vignettes, and narrative analysis

**DOI:** 10.3389/fneur.2025.1655455

**Published:** 2025-09-12

**Authors:** Giuseppe d’Orsi, Maria Teresa Di Claudio, Assunta Anna Soccio, Valentina Palumbo, Carmela Pia Ferro, Umberto Costantino, Raffaella Latino, Danilo Fogli

**Affiliations:** Neurology Unit, IRCCS Casa Sollievo della Sofferenza, San Giovanni Rotondo, Italy

**Keywords:** myoclonus, neurodegenerative disorders, video-EEG, dementia, basal ganglia degeneration, spinocerebellar degeneration, Lafora disease

## Abstract

**Purpose:**

Myoclonus—sudden, brief, involuntary muscle jerks—is a frequent and diagnostically challenging feature across neurodegenerative disorders. Unlike epileptic myoclonus, these manifestations rarely involve seizures; they often reflect extensive multisystemic pathology (cortical, subcortical, peripheral). Distinguishing neurodegenerative myoclonus from other movement disorders and pinpointing its origin is crucial for accurate diagnosis, prognosis, and tailored management. This narrative analysis explores the diverse phenomenology of myoclonus in neurodegenerative conditions, emphasizing how presentations often differ from typical epileptic myoclonus, which necessitates a nuanced diagnostic approach. We also highlight the indispensable role of video-electroencephalography (video-EEG) with polygraphy in this context.

**Methods:**

We synthesized existing literature on myoclonus in neurodegenerative disorders, complemented by illustrative clinical vignettes. The diagnostic utility of video-EEG with polygraphy was critically examined, focusing on its capacity to integrate motor behavior analysis with concurrent EEG and electromyographic (EMG) activity.

**Results:**

Myoclonus in neurodegenerative conditions (e.g., dementias, basal ganglia degenerations, spinocerebellar degenerations) presents with diverse phenomenology, often differing significantly from typical epileptic myoclonus. Video-EEG/polygraphy emerged as the gold standard, enabling precise characterization (focal, multifocal, generalized, rhythmicity, triggers) and localization of origin. Its adaptable protocols are fundamental for capturing the fluctuating, context-dependent nature of myoclonus in these progressive conditions, and for distinguishing pathological cortical excitability from subcortical or spinal origins.

**Conclusion:**

Video-EEG/polygraphy provides objective, real-time insights into the complex interplay of brain and muscle activity, profoundly refining our understanding of neurodegenerative myoclonus. This guides accurate differential diagnosis and informs effective patient care, proving essential for optimal patient management and prognosis in these challenging conditions.

## Introduction

Myoclonus, characterized by sudden, brief, involuntary muscle jerks resulting from active contractions or inhibitions, is a common neurological phenomenon that requires careful differentiation from other movement disorders (1). Thorough clinical observation, often combined with video-electroencephalography (video-EEG) and polygraphy, forms the cornerstone of its initial characterization and provides essential guidance. This provides essential guidance for subsequent neurophysiological and instrumental investigations aimed at a definitive diagnosis. Myoclonus can be classified from an *etiological perspective* into several categories: physiological, essential, epileptic, symptomatic, and psychogenic (2). A comprehensive diagnostic approach requires a multifaceted perspective that integrates this etiological framework with a detailed phenomenological assessment:

- *Physiological myoclonus* represents normal phenomena such as hiccupping or hypnic jerks (the sudden jerks felt when falling asleep) and does not indicate an underlying disease.
- *Essential myoclonus* is a rare, inherited condition characterized by a lifelong, chronic myoclonus that is not progressive and occurs in isolation, without other neurological symptoms.
- *Epileptic myoclonus* is directly related to epilepsy and often occurs as part of a seizure or as a dominant seizure type.
- *Symptomatic myoclonus* (also referred to as secondary myoclonus) arises as a manifestation of an underlying, often progressive, neurological or systemic disease.
- Finally, *psychogenic myoclonus* is a functional movement disorder without a clear organic cause, and its diagnosis is based on specific clinical features.

In parallel with this etiological framework, a systematic *clinical classification* is essential for a precise diagnosis. Myoclonus can be described based on its phenomenology:

- *Irregular vs. Rhythmic*: Irregular jerks are the most common form, while rhythmic myoclonus often suggests a specific subcortical or spinal origin.
- *At Rest vs. Action/Postural*: Myoclonus can occur spontaneously at rest or be triggered by voluntary movement (action myoclonus) or posture (postural myoclonus), a key feature in many neurodegenerative syndromes.
- *Reflex or Stimulus-Sensitive*: Myoclonus can be exacerbated or elicited by external stimuli, such as a sudden noise (startle) or a tactile sensation.

Within the etiological classification, myoclonus associated with neurodegenerative diseases falls under symptomatic myoclonus (2). Thus, alongside diverse etiologies such as metabolic, neoplastic, toxic, pharmacological, and autoimmune conditions, neurodegenerative causes represent a significant group, accounting for approximately 70% of symptomatic cases (3). This broad category includes dementias, basal ganglia and other neurodegenerative diseases, and spinocerebellar degenerations. Storage diseases are occasionally also included because, while their pathogenetic mechanisms may differ, the common final pathway is neurodegeneration. A classic example is progressive myoclonic epilepsies (PMEs), such as Lafora disease, a genetic disorder involving the progressive accumulation of insoluble, starch-like polyglucans (Lafora bodies), leading to progressive and fatal neurodegeneration (4). A key characteristic of myoclonus associated with neurodegenerative diseases (excluding PMEs) is the absence or rare co-occurrence of epileptic seizures, which are fundamental features of epileptic myoclonus. Conversely, there is often extensive multisystemic involvement of cortical, subcortical/basal ganglia, and even peripheral structures. Unlike epileptic myoclonus, which is defined by its strong association with seizures, the myoclonus associated with neurodegenerative diseases often reflects widespread, multisystemic pathology without overt seizures, necessitating a nuanced diagnostic approach based on clinical presentation and a systematic use of video-EEG/polygraphy. Herein, we present a narrative analysis of the various types of myoclonus associated with neurodegenerative diseases. This approach is particularly valuable due to the inherent phenotypic heterogeneity of myoclonus, allowing for the integration of diverse clinical and electrophysiological findings into a coherent understanding. We complement this analysis with illustrative clinical vignettes, typically studied using video-EEG/polygraphy, to enhance their recognition and differentiation in clinical practice.

### The role of video-EEG/polygraphy in myoclonus assessment

Given the inherent phenotypic, clinical, and etiological heterogeneity of myoclonus, video-EEG/polygraphy stands as the gold standard investigation due to its unique capacity to integrate motor behavior with concurrent neurophysiological activity (5). This technique integrates the analysis of motor behavior, specifically myoclonus, with concurrent electroencephalographic (EEG) and polygraphic activity, including electromyography (EMG). It serves as the foundation for advanced post-analysis techniques, such as back-averaging, which helps identify subtle cortical correlates preceding the myoclonic jerk. However, as an instrumental examination, its effective utilization critically hinges on a clinically guided, systematic, and rigorous methodological approach, applicable across all age groups. Comprehensive clinical evaluation, beginning with a detailed patient history and review of prior video-EEG/polygraphy recordings, is paramount. This initial assessment should dictate what to document and how and when to perform the recording. While the increasing availability of home videos can be informative, they must always be interpreted within a robust clinical context. Therefore, the strategic planning of video documentation, informed by the clinical picture, is essential to determine precisely what, how, and when to capture myoclonic events, ensuring relevant and impactful data.

### Methodology of video-EEG/polygraphy acquisition and analysis

Given the dynamic and heterogeneous nature of myoclonus in neurodegenerative disorders, the acquisition and analysis of video-EEG/polygraphic data require a targeted and adaptable methodological approach (5–10). There is no universal *‘standard’* protocol; rather, it is a dynamic strategy that evolves based on the individual clinical presentation and the natural history of the disease.

#### Essential electroclinical data

Accurate documentation is fundamental for correct diagnostic interpretation. During video-EEG/polygraphic recordings, it is imperative to capture:

- *Comprehensive Patient History:* A comprehensive patient history, encompassing symptom onset and progression, comorbidities, and pharmacological history, is crucial for contextualizing the myoclonus.
- *Prior Videos:* Any previous video recordings, whether clinical or home-based, are of great value for observing the evolution of myoclonus and its response to potential treatments.
- *Prior EEGs:* Previous EEG recordings provide context regarding baseline activity and the presence of any prior epileptiform abnormalities.
- *Specific Diagnostic Hypothesis:* Formulating a specific diagnostic hypothesis (e.g., cortical, subcortical, spinal myoclonus) based on initial clinical evaluation is imperative to guide targeted data acquisition and interpretation.

#### Instrumental parameters utilized

To adequately characterize myoclonus, video-EEG/polygraphy must utilize a combination of recordings:

- *Video-EEG:* Provides the temporal correlation between brain activity and visible motor manifestations, essential for identifying potential cortical correlates (e.g., cortical reflex myoclonus).
- *Electromyography (EMG):* The integration of EMG derivations from specific muscles is crucial. Typically, recordings include:
  - Bilateral deltoid muscles and flexor muscles for the limbs (e.g., biceps, wrist flexors) to assess symmetry and distribution.
  - Other muscle derivations may be added or substituted based on the observed phenomenology and distribution of myoclonus (e.g., facial muscles, leg muscles, truncal muscles).

### Evolving approach to data acquisition

Data acquisition is not static but an evolving process. Recording parameters (e.g., choice of muscles for EMG, type of provocative stimulation) must be adapted in real-time during the session, based on the clinical manifestations of myoclonus and the patient’s reactions. This “non-standard” approach reflects the necessity to personalize the diagnostic investigation.

### Adaptation based on disease history and therapy

Acquired data and recording methodology must be continuously re-evaluated and, if necessary, modified or supplemented with additional parameters based on the natural history of the patient’s disease. This also includes adapting the investigation to assess the response to specific therapies, allowing for the documentation of changes in myoclonus frequency, amplitude, or type in relation to pharmacological intervention. This methodological flexibility is indispensable for capturing the complexity of myoclonic presentations in neurodegenerative contexts.

### Neurophysiological classification of myoclonus

The diagnostic approach to myoclonus necessitates a precise neurophysiological classification, which provides essential clues regarding the site of origin and underlying pathophysiology. The gold standard for this assessment is video-EEG with polygraphic EMG, which allows for the detailed characterization of the myoclonus-related EMG burst and its temporal relationship with cerebral activity. Myoclonus can be broadly categorized into cortical, subcortical, spinal/segmental, and peripheral based on these findings (11).

- *Cortical Myoclonus:* This type originates from the cerebral cortex and reflects a focal or widespread increase in excitability within the sensorimotor cortex. This hyperexcitability can lead to sudden, involuntary muscle contractions. It is characterized by a very brief EMG burst (typically <50 ms). A key diagnostic feature is the presence of a time-locked cortical event, often a sharp wave or spike, preceding the EMG burst. This relationship is best identified through EEG–EMG back-averaging, a technique that averages multiple myoclonic jerks to reveal a subtle cortical correlate, known as the C-reflex. Cortical myoclonus is frequently stimulus-sensitive, triggered by sensory input, and is often associated with giant somatosensory evoked potentials (SEPs), which reflect heightened excitability of the sensorimotor cortex.
- *Subcortical Myoclonus*: Arising from subcortical structures like the brainstem or basal ganglia and is thought to result from the disinhibition of brainstem circuits, particularly the reticular formation. This myoclonus is not preceded by a clear cortical discharge. The associated EMG bursts are typically of longer duration (>50 ms) compared to cortical myoclonus. Back-averaging studies fail to show a preceding EEG correlate, confirming a non-cortical generator for the muscle jerk. Clinically, this type can be rhythmic or irregular and may manifest as a variety of syndromes, including reticular reflex myoclonus.
- *Spinal/Segmental Myoclonus:* This myoclonus originates from the spinal cord itself, affecting a single body segment. It is characterized by rhythmic or continuous EMG discharges in a specific myotome or group of muscles, with a burst duration that can be variable. Crucially, this type of myoclonus shows no associated EEG changes, as the neural activity is confined to the spinal cord. It is often a key feature in specific spinal pathologies.
- *Peripheral Myoclonus:* In rare cases, myoclonus can result from peripheral nerve hyperexcitability. This is characterized by repetitive discharges in a single motor unit or a small group of units, without any central nervous system correlate on EEG.

We will use this classification framework to more rigorously analyze and interpret the video-EEG/polygraphy findings presented in our clinical vignettes, providing a clearer link between the clinical presentation, the underlying pathophysiology, and the electrophysiological evidence. To provide a concise overview of these distinctions, we have summarized the key neurophysiological features of different myoclonus types in Table 1, which serves as a practical guide for their identification via video-EEG/polygraphy.

**Table 1:** Physiopathological and electrophysiological classification of myoclonus with the three main groups: cortical, subcortical, and spinal/segmental.

Feature	Cortical myoclonus	Subcortical myoclonus	Spinal/segmental myoclonus
Origin	Sensorimotor cortex	Basal ganglia, brainstem, thalamus	Spinal cord
EMG burst duration	Brief (<50 ms)	Longer (>50 ms)	Variable, often rhythmic/continuous
Associated EEG correlate	Time-locked cortical discharge (C-reflex) on back-averaging	No consistent preceding cortical correlate	None
Sensitivity to stimuli	Often stimulus-sensitive (reflex)	Variable	Not typically stimulus-sensitive
SSEP findings	May show giant somatosensory evoked potentials (SEPs)	Normal or non-specific changes	Normal
Clinical presentation	Focal, multifocal, or generalized jerks; often action-induced	Reticular reflex, generalized jerks, startle-induced	Rhythmic, focal jerks in a single body segment
Myoclonus type	Typically irregular	Can be rhythmic or irregular	Often rhythmic/pendular

While our analysis will primarily focus on the myoclonus associated with neurodegenerative diseases—a key subset of symptomatic myoclonus—it is crucial to consider the broader classification in the diagnostic process. Unlike epileptic myoclonus, which is defined by its strong association with seizures, the myoclonus we discuss here often reflects widespread, multisystemic pathology without overt seizures, necessitating a nuanced diagnostic approach based on clinical presentation and a systematic use of video-EEG/polygraphy.

## Myoclonus in neurodegenerative disorders

Symptomatic myoclonus (72%) is the most common type of myoclonus (1), followed by epileptic (17%) and essential myoclonus (11%). Myoclonus associated with neurodegenerative disorders is typically symptomatic. Unlike epileptic myoclonus, where the jerks are often a direct part of a seizure or a predominant seizure type, myoclonus in neurodegenerative conditions is rarely associated with overt epileptic seizures. Instead, it involves a broader range of cortical, subcortical (most commonly the basal ganglia), and even peripheral systems. Symptomatic myoclonus occurs in a wide range of neurodegenerative diseases, including dementias, basal ganglia disorders, and spinocerebellar degenerations (Supplementary Figure 1). While seizures may occasionally be a significant part of a patient’s overall illness, they generally do not represent the primary myoclonus phenotype as seen in epileptic myoclonus. Other neurological systems are typically affected, potentially involving cognition, parkinsonism, ataxia, sleep dysfunction, autonomic symptoms, upper motor neuron findings, and even peripheral nerve dysfunction (2).

## Dementias

Myoclonus can commonly appear in individuals with chronic forms of dementia (such as Alzheimer’s disease) and rapidly progressive dementias (RPDs) like prion diseases (e.g., Creutzfeldt-Jakob disease, CJD) (12).

### Alzheimer’s disease

In Alzheimer’s disease (AD), the cumulative probability of developing myoclonus can be as high as 42% (13). The usual presentation involves small, multifocal distal jerking, but widespread or generalized jerks may also be present (2, 3). When the myoclonus in AD is large and widespread, it may be confused with CJD, given the prominent myoclonus often seen in prion diseases (3). Although myoclonus in AD often develops in the later stages of the disease (14), an earlier age of disease onset, faster progression, or familial causes of AD are associated with myoclonus appearing earlier and with a higher incidence (13).

#### Clinical vignette 1

An 79-year-old patient presented with a slowly progressive onset of cognitive and behavioral issues starting at the age of 67, including significant memory impairment, aphasia, and executive dysfunction, accompanied by a gradual loss of autonomy. Brain MRI revealed cerebral atrophy, predominantly affecting the temporal regions. Cerebrospinal fluid biomarkers were conclusive for AD. At the age of 81, the patient developed irregular, rapid, and brief myoclonic jerks. These jerks were primarily at rest but could also be action-induced, affecting the upper limbs, usually distally, and occurring both unilaterally and bilaterally. Video-EEG/polygraphy demonstrated, within the context of a mild diffuse slowing of background activity, the presence of isolated and diffuse epileptiform abnormalities, usually with a corresponding rapid muscular potential in the explored muscle derivations. This electrophysiological pattern, with a cortical correlate preceding the EMG burst, suggests a cortical origin for the myoclonus, consistent with widespread cortical dysfunction in advanced AD (see Supplementary Video 1, patient 1).

#### Clinical vignette 2

An 83-year-old patient presented with a long-standing history of cognitive decline and behavioral disturbances that began at the age of 72. The cognitive impairment was characterized by progressive memory loss, disorientation, and executive dysfunction. Clinical, neuropsychological, instrumental, and laboratory examinations were conclusive for AD. In the last 3 years, the patient developed irregular, asymmetrical, and focal myoclonic jerks predominantly affecting the upper limbs. These jerks were typically action-induced and sometimes presented as massive movements, contributing to a decline in motor function (see Supplementary Video 1, patient 2). The video-EEG/polygraphy in this patient, while not showing a clear cortical correlate in all instances, demonstrated the variable and complex nature of myoclonus in advanced AD, with findings suggestive of both cortical and subcortical origins.

### Progressive myoclonic epilepsy in Down syndrome

Beyond the age of 40, nearly all patients with Down syndrome (DS) develop AD due to overexpression of the amyloid precursor protein gene on chromosome 21. A subset of these patients may develop a form of PME characterized at onset by the appearance of focal and multifocal, sometimes massive myoclonic jerks, usually evident upon morning awakening, more often following a generalized tonic–clonic seizure (15). Video-EEG/polygraphy shows the presence of diffuse, rapid polyspike-wave epileptiform abnormalities associated with myoclonic-type muscular potentials in the explored muscle regions. In the natural history of the disease, these patients develop cortical and subcortical myoclonus, gait disturbance, and severe dementia, completing the syndromic picture of PME.

#### Clinical vignette

**Figure 1:**
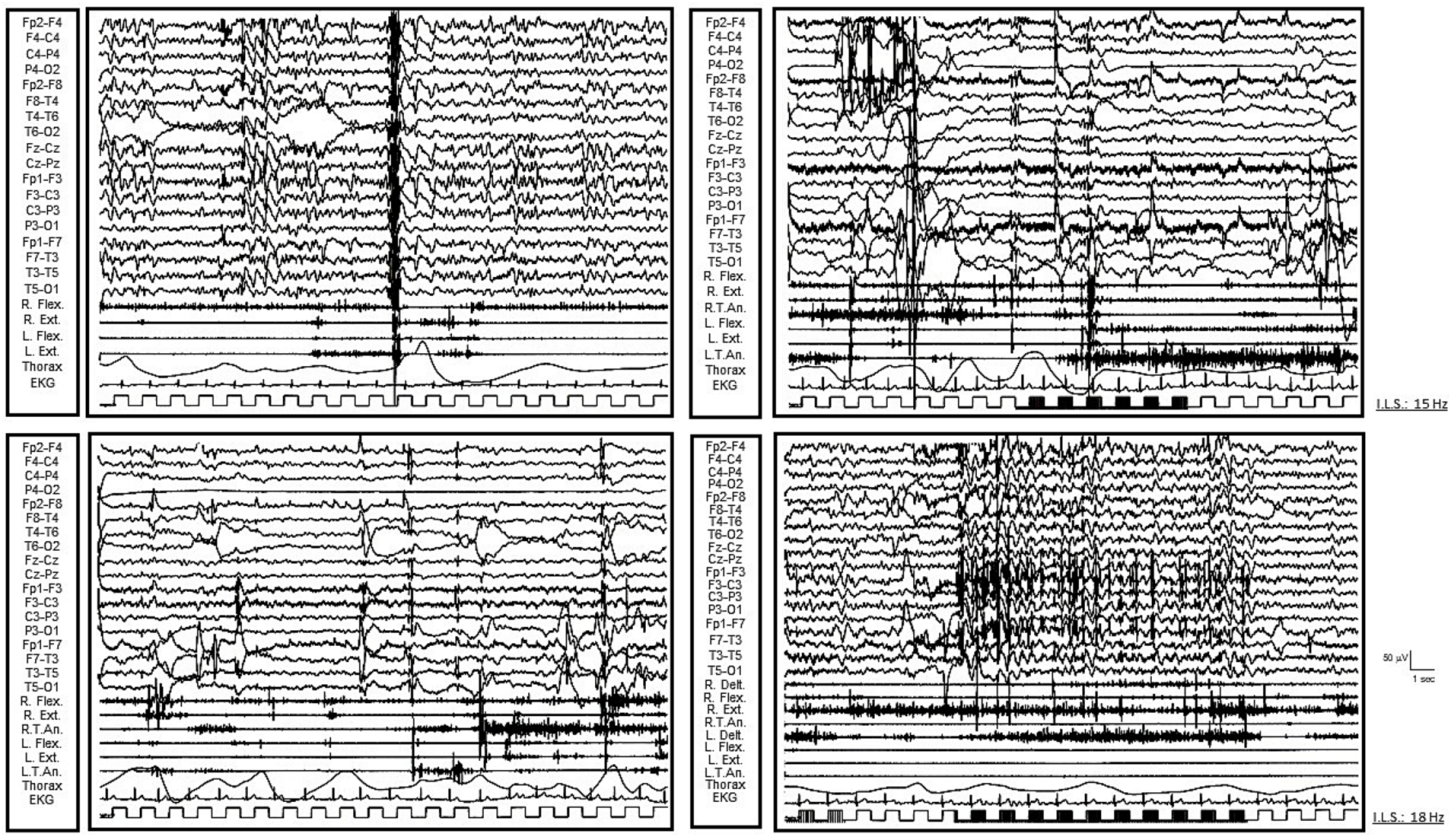
Video-EEG/polygraphic features of progressive myoclonic epilepsy in down syndrome (R. and L. Delt.: right and left deltoid muscles; R. and L. Flex.: right and left wrist flexor muscles; R. and L. Ext: right and left wrist extensor muscles; R. and L. T. Ant.: right and left tibialis anterior muscles; Thorax: thoracic respiration; EKG: electrocardiogram). *Left Panel:* The upper and lower sections show diffuse sharp-wave and polyspike-wave complexes over a globally slowed background activity. These are linked to shock-like myoclonic muscular potentials, mainly affecting the upper limb muscles. *Right Panel:* The upper section shows that intermittent photic stimulation (IPS) at 15 Hz causes a diffuse photomyoclonic response in Patient 1. The lower section illustrates that IPS at 18 Hz triggers a focal photomyoclonic response in the right upper limb of Patient 2.

Two DS patients, with an average age of 45, have experienced a progressive loss of independence over several years. This decline was characterized by gradual cognitive deterioration, including disorientation, confusion, loss of interest, and apathy, consistent with Alzheimer’s disease. Upon morning awakening, they exhibit a generalized tonic–clonic seizure followed by approximately 1 h of bilateral, asymmetrical, and irregular myoclonus (typically affecting the upper limbs). Video-EEG polygraphy reveals epileptiform abnormalities, specifically diffuse spike-and-polyspike-and-slow-wave complexes, which are occasionally associated with rapid myoclonic potentials in the examined muscle groups. This finding suggests a cortical–subcortical origin for the myoclonus, a hallmark of progressive myoclonic epilepsy (PME). Intermittent photic stimulation (IPS), at medium photic stimulation frequencies, elicits a photo-myoclonic response, further indicating a heightened cortical excitability (see Supplementary Video 2, and Figure 1).

### Familial Alzheimer’s Disease

Familial Alzheimer’s Disease (FAD) is a rare, inherited form of AD characterized by an early age of onset, typically before 65 years. Unlike sporadic AD, FAD is directly caused by autosomal dominant mutations in the *APP*, *PSEN1*, or *PSEN2* genes, leading to abnormal amyloid-beta processing and subsequent neurodegeneration. Clinically, FAD presents with progressive cognitive decline similar to sporadic AD, including memory loss, executive dysfunction, and language difficulties. However, the progression is often more rapid. A key differentiating factor is the earlier onset, frequently in the 40s or 50s. Crucially, certain FAD forms, especially those linked to PSEN1 mutations, can present with additional, atypical neurological features not commonly seen in sporadic AD. Among these, myoclonus is a notable symptom that can appear early in the disease course. Other atypical features may include seizures, spasticity, gait disturbances, and pronounced psychiatric symptoms (16).

#### Clinical vignette

**Figure 2:**
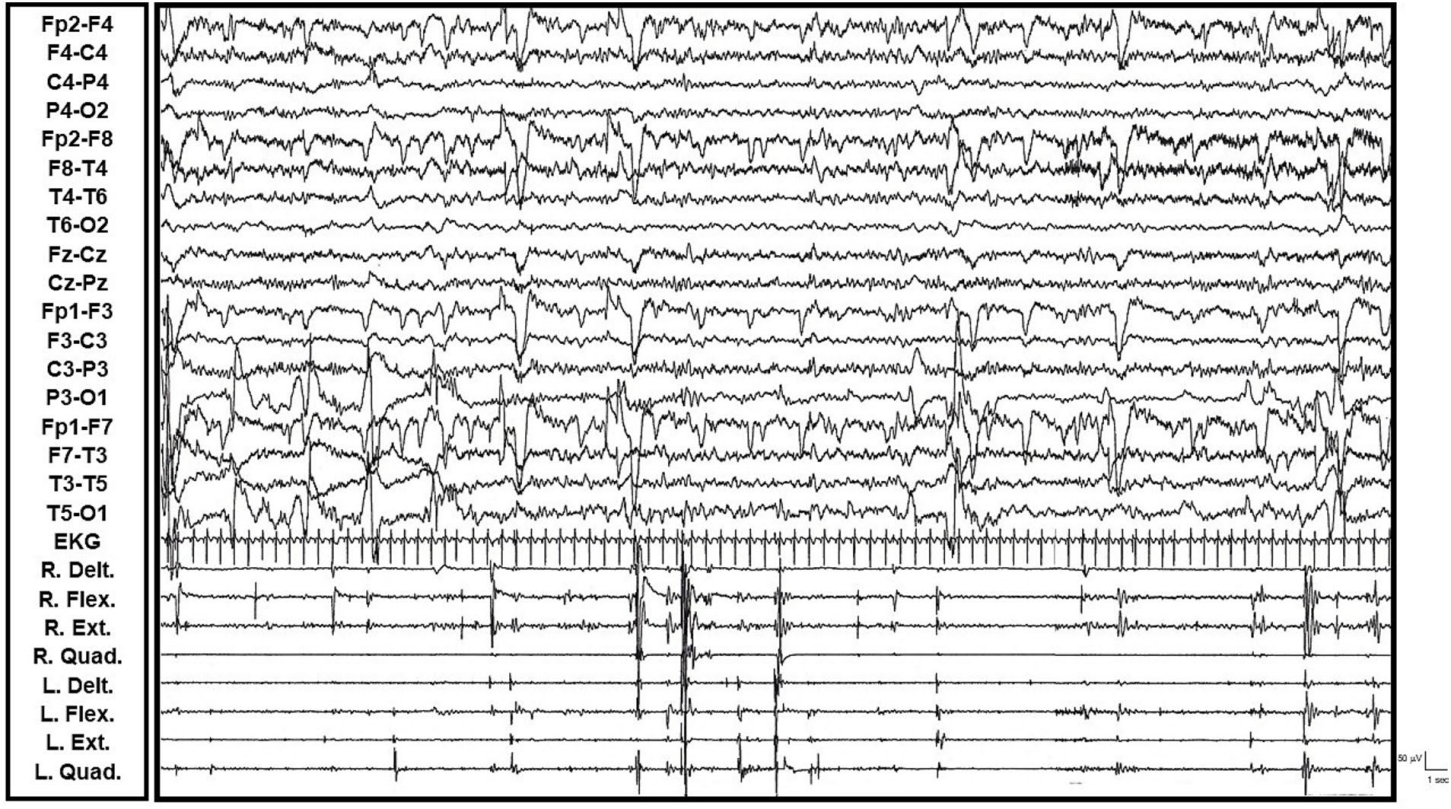
Video-EEG/polygraphic features of Familial Alzheimer’s Disease (PSEN1 gene mutation) (R. and L. Delt.: right and left deltoid muscles; R. and L. Flex.: right and left wrist flexor muscles; R. and L. Ext: right and left wrist extensor muscles; R. and L. Quad.: right and left quadriceps femoris muscles; EKG: electrocardiogram). Multifocal, parcellar, and massive, synchronous and asynchronous myoclonic jerks observed in all four limbs during periods of quiet wakefulness and fluctuating vigilance. These jerks were not consistently associated with epileptiform abnormalities (even after back-averaging) and occurred within a context of global slowing of background activity.

A 36-year-old patient, whose sibling was affected by AD, presented with a slowly progressive gait disturbance and cognitive decline, including aphasia, agnosia, and apraxia, starting at age 33. Concurrently, the patient developed irregular, focal, and multifocal myoclonic jerks. These jerks were present at rest and were exacerbated by movement and changes in posture (action-induced myoclonus). Video-EEG/polygraphy documented multifocal myoclonic jerks which were not always associated with epileptiform abnormalities (even after back-averaging). The myoclonus occurred within a context of global slowing of background activity, suggesting both cortical and subcortical origins due to the lack of a consistent cortical correlate. Genetic evaluation revealed a heterozygous missense mutation in the PSEN1 gene (exon 7; nucleotide: c.689 T > C; amino acid: p.Met233Thr), confirming a diagnosis of Familial Alzheimer’s Disease (see Supplementary Video 3, and Figure 2).

These cases collectively emphasize that while cognitive impairment remains the central diagnostic criterion for AD, the presence and specific characteristics of myoclonus can offer valuable insights into the underlying disease mechanisms and the affected neuroanatomical substrates. The varying EEG correlates and clinical evolution in these cases suggest that myoclonus in AD can arise from a spectrum of pathophysiological processes. These processes range from focal cortical hyperexcitability in some presentations to a more widespread cortical and subcortical dysfunction influenced by genetic factors and disease progression. Recognizing these diverse presentations is crucial for accurate diagnosis, differential considerations, and a more comprehensive understanding of the complex neurobiology of AD.

### Creutzfeldt-Jakob disease

CJD is the emblematic example of rapidly progressive dementia associated with myoclonus. Sporadic CJD accounts for approximately 90% of all prion disease cases, with myoclonus being particularly prevalent (up to 97%) in patients carrying the methionine/methionine or methionine/valine genotype at codon 129 (17). While the hallmark of CJD is rapidly progressive dementia, myoclonus is a highly characteristic and prevalent feature of the disease. It can occur at rest, during voluntary muscle activation, or in response to various stimuli (e.g., acoustic, visual, and tactile). Typically, myoclonus becomes more prominent in the terminal stages of the disease, presenting as massive, focal, or multifocal jerks. This often manifests as periodic myoclonus, occurring continuously with a periodicity of 0.6–1.2 Hz. Frequent clinical and video-EEG/polygraphic evaluations are crucial. Myoclonus can sometimes have a fluctuating course, and it often needs to be specifically sought out and provoked during recordings through examination and stimulation with various sensory inputs. Similar to typical periodic epileptiform discharges on EEG, CJD-associated myoclonus tends to reduce or disappear during fluctuations in the level of vigilance, often reappearing after external stimulation, or following an apnea or upon to an alert state (18, 19). This EEG/polygraphic pattern is an expression of the underlying neurodegenerative pathology, therefore aggressive symptomatic therapy is not justified. This highlights the importance of accurate diagnosis and the potential for misclassification in complex neurological conditions, particularly differentiating rapidly progressive dementias with myoclonus from true non-convulsive status epilepticus (NCSE). For CJD, key differentiating features from widespread AD myoclonus often include rapid progression, the presence of characteristic periodic sharp wave complexes on EEG, and specific CSF biomarkers like 14-3-3 protein or real-time quaking-induced conversion.

#### Clinical vignette

**Figure 3:**
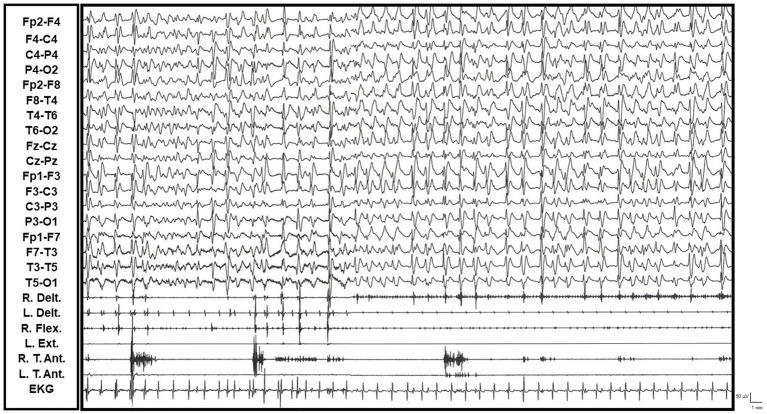
Video-EEG/polygraphic features of Creutzfeldt-Jakob disease (R. and L. Delt.: right and left deltoid muscles; R. and L. Flex.: right and left wrist flexor muscles; R. and L. T. Ant.: right and left tibialis anterior muscles; EKG: electrocardiogram). Rhythmic and arrhytmic, subcontinuous myoclonus, diffuse, predominantly affecting the right limbs, especially the right upper limb. Myoclonus was exacerbated by movement and frequently, but not always, coincided with periodic diffuse epileptiform discharges on EEG.

A 56-year-old patient presented with a progressive cognitive decline of approximately 4 months’ duration. This decline was characterized by rapidly progressive cognitive impairments (disorientation, attentional and concentration deficits, loss of autonomy) and behavioral disturbances (insomnia, restlessness, complex visual hallucinations). In recent weeks, the patient developed rhythmic, subcontinuous myoclonus in all four limbs, which was exacerbated by movement and showed a predominance in the right upper limb. These jerks often coincided with periodic generalized epileptiform discharges on EEG, a hallmark of CJD. While tactile stimulation and movement accentuated the myoclonus, there was no response to auditory stimuli. Genetic testing revealed a methionine/methionine genotype at codon 129 of the PRNP gene (see Supplementary Video 4 and Figure 3).

## Basal ganglia degenerations

### Corticobasal degeneration

Myoclonus is found in a high percentage (55–93%) of individuals with corticobasal syndrome (CBS). CBS is often considered a bridge between forms of dementia and basal ganglia degeneration, exhibiting a complex interplay of cognitive and motor features due to both cortical and subcortical pathology. However, myoclonus is present in only 27% of those with pathologically confirmed corticobasal degeneration (CBD) (20). This myoclonus is typically action-induced and stimulus-sensitive, with a focal distribution often involving the dystonic limb. This pattern suggests the uneven cortical and subcortical brain abnormalities seen on neuroimaging. Neurophysiological studies consistently show the involvement of a subcortical network (21). Although CBD myoclonus shares characteristics with cortical myoclonus, such as short-duration, hypersynchronous bursts of electromyographic (EMG) activity in agonist and antagonist muscles, it also presents key distinctions. Unlike classic cortical reflex myoclonus, CBD-related myoclonus is often not associated with enlarged somatosensory evoked potentials (SEPs) or preceding cortical discharges in back-averaged EEG recordings. It may also exhibit a slightly shorter reflex latency, suggesting distinct pathophysiological mechanisms (22, 23). The severity and the challenge in treating this myoclonus in CBD have led to investigations into novel therapeutic approaches. Levetiracetam, for instance, has shown promise in suppressing myoclonic activity in some cases, possibly by modulating cortical excitability (24).

#### Clinical vignette

A 64-year-old patient presented with a gradual onset of left-sided motor difficulties, including the sensation of an alien limb, accompanied by ideational slowing and progressive cognitive decline. Concurrently, the patient experienced brief episodes of loss of consciousness preceded by an ascending epigastric sensation, consistent with focal seizures. Electroencephalography (EEG) revealed epileptiform abnormalities predominantly in the left temporal derivations. Brain magnetic resonance imaging (MRI) showed diffuse cerebral atrophy. Neurological examination revealed a left pyramidal syndrome and both positive and negative myoclonus, which was irregular, action-induced, and stimulus-sensitive, triggered by touch and emerging distally in the patient’s left upper limb. Treatment with levetiracetam 1,000 mg/day resulted in a significant reduction in the focal seizure episodes, while the myoclonus showed only a slight improvement. Lacosamide up to 200 mg/day distinctly worsened the patient’s myoclonus. Over a three-year clinical follow-up, the patient’s motor issues progressively worsened, leading to a bedridden state, characterized by focal, typically reflex, myoclonus in the left upper limb, associated with global cognitive deterioration (see Supplementary Video 5).

### Parkinson’s disease (PD)

In Parkinson’s Disease (PD), small, non-synchronous myoclonus that affects posture or action can occur bilaterally. This can sometimes be a side effect of L-Dopa/amantadine treatment (25). A subtle form of cortical myoclonus has been noted in about 5% of PD patients without dementia (26). Electrophysiological studies in some cases have revealed evidence of sensorimotor cortex dysfunction, even when the primary pathology is more widespread (27). Clinically, this type of myoclonus can be hard to tell apart from a tremor, often requiring EMG for a clear diagnosis to differentiate the rhythmic, oscillatory nature of tremor from the sudden, shock-like contractions of myoclonus. Finally, a potential link between elevated levels of *α*-synuclein in the primary motor cortex and the presence of cortical myoclonus in PD, even in the absence of significant Alzheimer’s disease pathology, has been reported (28).

### Multiple system atrophy (MSA)

In Multiple System Atrophy (MSA), myoclonus patterns can differ between its subtypes. Reflex myoclonus, which is triggered at rest by various stimuli (e.g., tactile, auditory), is more commonly observed in patients with the cerebellar type of MSA (MSA-c) than in those with the parkinsonian type (MSA-p) (29). This likely reflects the more prominent involvement of cerebellar circuits in MSA-c, which can modulate cortical excitability. Conversely, distal postural/action myoclonus is frequently seen in MSA-p patients and is believed to originate in the cortex (30). This cortical myoclonus in MSA-p might contribute to the difficulty in performing fine motor tasks and maintaining posture, further exacerbating the parkinsonian features. The presence and characteristics of myoclonus in MSA can thus offer valuable clinical clues, contributing to the differentiation between MSA subtypes and from other parkinsonian syndromes.

### Huntington’s disease

While myoclonus is not a primary defining feature of Huntington’s disease (HD), it can be a clinically significant symptom, particularly in the later stages or in the less common juvenile form (31, 32). HD is a progressive neurodegenerative disorder characterized primarily by chorea, but other hyperkinetic movements can also manifest. Myoclonus, in this context, is typically action-induced and postural, and it is more often observed in the face, trunk, and proximal limbs. Neurophysiological studies suggest that the myoclonus in HD is primarily cortical in origin. This is thought to result from a loss of inhibitory control within the striato-thalamo-cortical motor circuits, a key pathological pathway in HD. This disinhibition leads to a heightened excitability of the motor cortex, which can be evidenced by the presence of a C-reflex on back-averaging and, in some cases, by enlarged somatosensory evoked potentials. While the presence of myoclonus in HD is less common than chorea, its correct identification is crucial for a complete clinical picture and for guiding symptomatic management, which may include antiepileptic drugs that modulate cortical excitability.

## Spinocerebellar degenerations

In this third group of disorders, ataxia is the primary and often most debilitating symptom, frequently presenting as slowly progressive and with a variable age of onset. The Syndrome of Dyssynergia Cerebellaris Myoclonica, also known as Ramsay Hunt syndrome (RHS), exemplifies this by being characterized as an idiopathic, progressive myoclonic ataxia. While it shares clinical similarities with PMEs, progressive myoclonic ataxia (PMA) is distinguished by a more prominent ataxia and less frequent, typically less severe, seizures compared to PME. This distinction is crucial, as PME often presents with severe, drug-resistant epilepsy and more profound cognitive decline, while PMA cases may have only mild cognitive impairment or infrequent, treatment-responsive seizures (33, 34). The myoclonus observed in these conditions is often multifocal, segmental, or occasionally generalized. It is characteristically non-rhythmical and can be accentuated or induced by movement (action myoclonus), which can significantly impair motor coordination and daily activities. In some instances, it’s reflex, triggered by auditory or visual stimuli, indicating a heightened excitability of specific neural circuits. EEG studies may reveal isolated or generalized polyspike-wave bursts, although distinct epileptic discharges are less consistently prominent than in PME. Myoclonus is reported to be of cortical origin in 70% of PMA cases (33), suggesting that despite the primary cerebellar pathology, a disinhibition of cortical circuits, potentially due to altered cerebello-thalamo-cortical pathways, plays a significant role in its generation (34, 35). The loss of cerebellar inhibitory control is a leading hypothesis for the enhanced excitability of the sensorimotor cortex observed in these conditions (21). Differential diagnoses for progressive myoclonic ataxia are broad and include both genetic and acquired etiologies, such as coeliac disease, mitochondrial disorders [particularly Myoclonic Epilepsy with Ragged Red Fibers (MERRF)], various forms of spinocerebellar degeneration (SCA2, SCA3, and SCA7), Dentatorubral-pallidoluysian atrophy (DRPLA), post-anoxic encephalopathy, Late-onset inherited metabolic encephalopathies, Ataxia-telangiectasia, Friedreich’s ataxia (23, 36). The clinical and electrophysiological assessment of myoclonus, in conjunction with thorough genetic testing and neuroimaging, is essential for accurate diagnosis and prognostication within this complex group of progressive myoclonic ataxia syndromes. Understanding the precise origin and characteristics of myoclonus is vital for tailoring symptomatic treatments, which often include antiepileptic drugs like valproate, levetiracetam, or clonazepam, even in the absence of overt seizures (37).

### Clinical vignette 1

A 60-year-old man presented with a gradual onset, around age 50, of postural instability and gait unsteadiness. He experienced traumatic spontaneous falls often triggered by sudden jerks in the lower limbs. Concurrently, he developed irregular, multifocal jerks in the upper limbs, occurring both at rest, during posture, and with action. These events were accentuated by emotionally significant situations. There was no evidence of cognitive impairment. Video-EEG polygraphy revealed diffuse epileptiform abnormalities, both isolated and in brief bursts, which were accentuated during sleep. These were sometimes associated with rapid muscle potentials consistent with myoclonus. The lack of a consistent cortical correlate preceding the EMG bursts suggests a subcortical origin, likely stemming from the altered cerebello-thalamo-cortical pathways. Brain MRI was unremarkable. Extensive laboratory, muscle and skin biopsy, and exome sequencing investigations did not reveal clear alterations. Therapeutic attempts with clonazepam and levetiracetam provided partial control of the myoclonic symptomatology (see Supplementary Video 6).

### Clinical vignette 2

**Figure 4:**
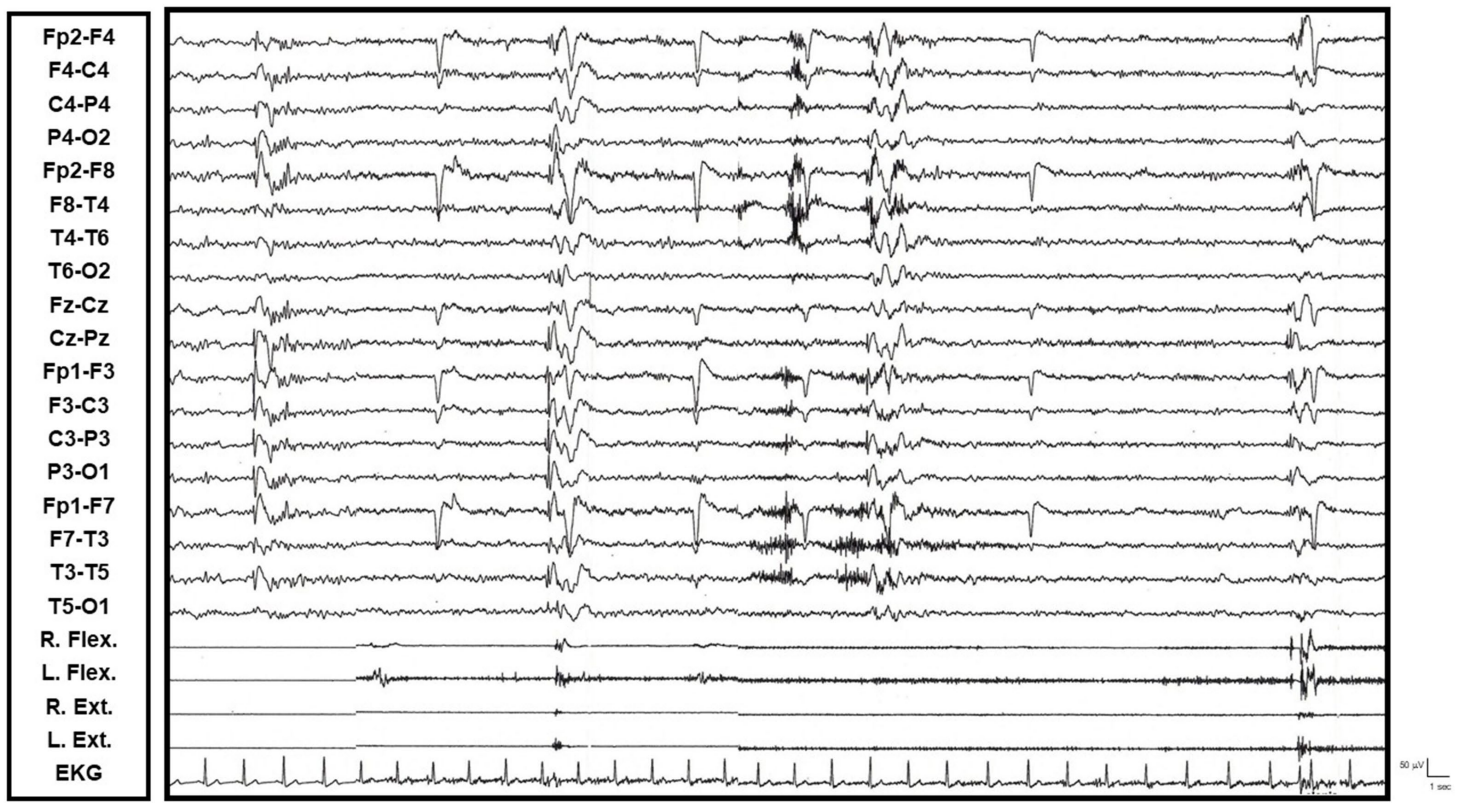
Video-EEG/polygraphic features of progressive myoclonic ataxia (R. and L. Flex.: right and left wrist flexor muscles; R. and L. Ext: right and left wrist extensor muscles; EKG: electrocardiogram). Isolated, diffuse epileptiform abnormalities, predominant over the left anterior derivations, are sometimes associated with a rapid, shock-like myoclonic-type muscle potential evident in the explored muscle districts.

A 45-year-old man presented with a gradual onset, around age 20, of postural instability and gait unsteadiness. This was associated with irregular, multifocal myoclonic jerks in the upper limbs, usually occurring during posture and with action. These events were accentuated by emotionally significant situations. There was no evidence of cognitive impairment, despite an associated anxiety-depressive disorder with behavioral disturbances. Video-EEG polygraphy revealed diffuse epileptiform abnormalities, both isolated and in brief bursts, which were sometimes associated with myoclonic jerks. The myoclonus was found to be cortical–subcortical in origin, as it lacked a consistent cortical correlate on back-averaging. Brain MRI showed mild cerebral atrophy. Extensive laboratory, muscle and skin biopsy, and genetic (exome) investigations did not reveal clear alterations. Therapeutic attempts with clonazepam, levetiracetam, and perampanel provided partial control of the myoclonic symptomatology. However, perampanel, up to 8 mg/day, exacerbated psychiatric and behavioral issues (see Figure 4).

### Progressive myoclonic epilepsies

Myoclonus, while not strictly classified as symptomatic myoclonus but rather as epileptic myoclonus, is a defining characteristic of PMEs (38). These rare, inherited neurodegenerative disorders are marked by a progressive escalation of myoclonus, manifesting as brief, involuntary muscle jerks frequently provoked by action, posture, or sensory input. Unlike benign myoclonus, the myoclonus in PMEs is typically debilitating, significantly hindering daily functions. PMEs are commonly associated with other neurological deficits, including generalized tonic–clonic seizures, ataxia, and cognitive deterioration. The underlying pathology often involves extensive neuronal degeneration, affecting various brain regions, notably the cerebellum and cerebral cortex. The myoclonic jerks in PMEs are frequently cortical in origin, arising from hyperexcitable motor cortical areas. This can be evidenced on EEG by giant somatosensory evoked potentials or polyspike-and-wave discharges precisely correlated with the myoclonic jerks. Nevertheless, subcortical and brainstem generators can also contribute to the myoclonus in certain PME subtypes. Lafora disease (LD) is a type of a PME, characterized by the relentless and progressive accumulation of insoluble polyglucosan bodies, culminating in fatal neurodegeneration (39). LD underscores how diverse genetic and pathological mechanisms can converge to produce the devastating common phenotype of progressive myoclonus and neurological decline observed in PMEs.

### Clinical vignette

**Figure 5:**
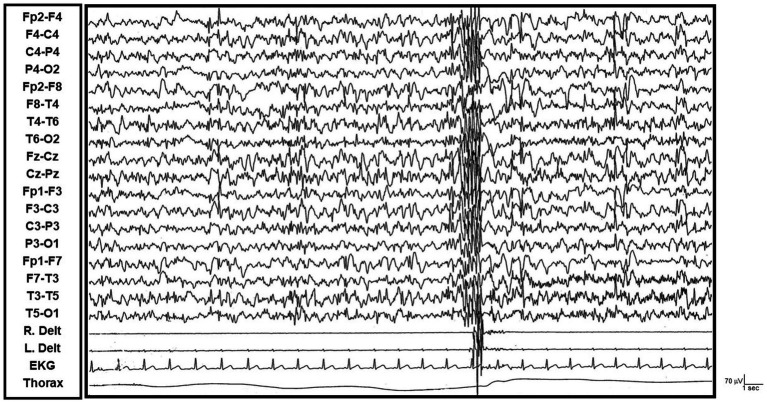
Video-EEG/Polygraphic features of Progressive Myoclonic Epilepsy (Lafora disease, late stage). (R. and L. Delt.: right and left deltoid muscles; Thorax: thoracic respiration; EKG: electrocardiogram). A global slowing of background activity is superimposed with frequent, rapid, polyspike epileptiform abnormalities. These are sometimes associated with massive myoclonic jerks predominantly affecting the proximal upper limbs.

A 18-year-old male presented with a history of sudden, irregular myoclonic jerks of the upper limbs since age 13, typically occurring upon awakening. Initial EEG revealed diffuse epileptiform abnormalities. Treatment with levetiracetam up to 1,000 mg/day achieved clinical control, leading to an initial misdiagnosis of Juvenile Myoclonic Epilepsy (JME). Approximately 2 years later, his myoclonic seizures worsened, becoming pharmacoresistant and present at rest, during action, and in postural positions, affecting all four limbs. Concurrently, he developed monthly generalized tonic–clonic seizures, gait disturbance, and cognitive/ideomotor slowing. Video-EEG polygraphy demonstrated a global slowing of background activity superimposed with frequent, rapid, polyspike epileptiform abnormalities. Disorganized sleep architecture also revealed epileptiform abnormalities predominantly over the bilateral posterior derivations. The frequent polyspike activity, often correlating with the myoclonic jerks, is highly suggestive of a cortical origin for the myoclonus. Next-generation sequencing (NGS) identified a pathogenic mutation in the EPM2A gene (encoding laforin) on chromosome 6, confirming a diagnosis of Lafora Disease (LD) (see Figure 5).

### Therapeutic strategies for myoclonus in neurodegenerative disorders

The therapeutic management of myoclonus in neurodegenerative disorders is often complex, as its primary goal is symptomatic control rather than curing the underlying pathology. The choice of medication is critically dependent on the myoclonus’s neurophysiological origin, which is precisely what video-EEG/polygraphy helps determine (2, 40, 41). Antiseizure medications are considered the first-line treatment, though their efficacy varies significantly across different syndromes (Table 2).

**Table 2:** Myoclonus therapy in neurodegenerative disorders with the three main groups: cortical, subcortical, and spinal/segmental.

Myoclonus type	Primary origin	First-line medications	Other effective medications	Important notes
Cortical	Sensorimotor cortex	Levetiracetam, piracetam, valproate	Brivaracetam, Perampanel, Zonisamide	Effective at reducing cortical hyperexcitability. Be mindful of cognitive side effects.
Subcortical	Brainstem, basal ganglia	Clonazepam	Gabapentin, Pregabalin, Baclofen	Effective for rhythmic or startle-induced myoclonus. May cause sedation.
Spinal/segmental	Spinal CORD	Clonazepam, valproate	Baclofen, botulinum toxin	Often difficult to treat. Botulinum toxin can be helpful for focal forms.

#### Pharmacological management by myoclonus origin

- *Cortical Myoclonus:* For myoclonus originating in the cerebral cortex, the goal is to reduce cortical hyperexcitability. Medications such as Levetiracetam and Piracetam are often the preferred choices. Valproate is another effective option, particularly for generalized myoclonus.
- *Subcortical and Reticular Myoclonus:* When the myoclonus originates from subcortical structures like the brainstem, medications that enhance the action of GABA (gamma-aminobutyric acid) are typically more effective. Clonazepam is considered a first-line drug due to its potent action on GABA-A receptors.
- *Spinal Myoclonus:* Originating from the spinal cord, this type of myoclonus can be particularly challenging to treat. Clonazepam and Valproate are often used. Additionally, muscle relaxants like Baclofen can be beneficial. For highly focal or segmental spinal myoclonus, Botulinum Toxin injections may be considered for targeted symptomatic relief.

#### Considerations for treatment

It is crucial to approach therapeutic management with caution. The efficacy of these medications must be evaluated individually, as they can sometimes worsen existing motor or cognitive symptoms in this patient population. Therefore, pharmacological management requires a highly individualized approach, carefully balancing the need to control myoclonus with the preservation of residual cognitive and motor functions.

## Conclusion

Myoclonus, a complex and diverse neurological phenomenon, frequently complicates the clinical picture of various neurodegenerative diseases (Table 3). As we have explored, its characteristics, electrophysiological signatures, and underlying pathologies vary significantly across conditions like dementias, basal ganglia degenerations, spinocerebellar degenerations, and also in PMEs, which primarily manifest as epileptic myoclonus. This inherent heterogeneity underscores the critical need for a meticulous and nuanced diagnostic approach. In navigating the complexities of myoclonus in neurodegenerative disorders, video-EEG/polygraphy emerges as the gold-standard investigation. This powerful tool allows for the simultaneous integration of motor behavior analysis with concurrent EEG and EMG activity. As demonstrated by the illustrative cases, video-EEG/polygraphy is not merely a confirmatory test but an essential compass for clinical navigation. Its value lies in:

- Precise Characterization: It enables the detailed characterization of myoclonus, capturing its focal, multifocal, or generalized nature, its rhythmicity (or lack thereof), and its triggers (e.g., movement, sensory stimuli).
- Localization of Origin: By correlating muscle jerks with specific brain activity, video-EEG/polygraphy helps pinpoint the origin of myoclonus (e.g., cortical, subcortical), which is crucial for diagnostic hypothesis generation.
- Differentiation from Mimics: It allows for the accurate differentiation of myoclonus from other movement disorders and, importantly, helps distinguish between epileptic and non-epileptic forms—a key factor in guiding management and prognosis. For instance, distinguishing the myoclonus of CJD from that in advanced AD or true NCSE relies heavily on these integrated findings.
- Dynamic Assessment: The adaptable nature of video-EEG/polygraphy protocols—tailoring muscle derivations, provocative stimuli, and recording duration—is vital for capturing the often fluctuating and context-dependent nature of myoclonus in neurodegenerative diseases. This methodological flexibility is indispensable for documenting changes in response to disease progression or therapeutic interventions.

**Table 3:** Comparative features of myoclonus in neurodegenerative disorders.

Disorder	Phenomenology	Triggers	EEG correlates	Anatomical origin	Therapeutic response
Alzheimer’s disease	Irregular, multifocal, distal jerks; can be widespread.	At rest, action-induced.	Mild diffuse slowing; isolated epileptiform abnormalities.	Cortical, subcortical.	Partial response to antiseizure medications.
Down syndrome	Myoclonus, often as part of late-onset myoclonic epilepsy (LOMEDS) in patients with Alzheimer’s dementia.	Sensory stimulation (e.g., upon awakening).	Diffuse slowing of background activity; generalized polyspike-wave discharges, especially in the morning.	Cortical (related to hyperexcitability).	Variable response; Levetiracetam and Valproate are first-line. Perampanel can be an effective second-line option.
Creutzfeldt-Jakob disease	Rhythmic, subcontinuous myoclonus.	Spontaneous, movement, sensory stimulation.	Periodic Sharp Wave Complexes (PSWC).	Predominantly subcortical (brainstem).	Poor. Symptomatic management.
Corticobasal degeneration	Focal, action-induced, stimulus-sensitive jerks.	Voluntary movement, touch.	Absence of giant SEPs or consistent cortical discharges.	Subcortical network (cortico-basal ganglia).	Variable; Levetiracetam may be effective.
Multiple system atrophy	Reflex myoclonus (MSA-c); Postural/action myoclonus (MSA-p).	Sensory stimuli (MSA-c).	No clear EEG correlate.	Subcortical (cerebellar circuits) or cortical.	Often poor; symptom-specific.
Huntington’s disease	Irregular, variable myoclonus, often part of chorea.	Spontaneous, at rest.	Normal or non-specific slowing.	Subcortical (basal ganglia).	Often difficult to treat. Chorea medications (e.g., VMAT2 inhibitors) may help.
Progressive myoclonic epilepsies (PME)	Progressive, severe myoclonus; photosensitive.	Sensory stimuli, especially photic stimulation.	Diffuse spike-and-polyspike-and-slow-wave complexes.	Cortico-subcortical.	Initial response to valproate/levetiracetam, but progress

In summary, while the clinical presentation provides the initial roadmap, video-EEG/polygraphy acts as the indispensable instrument that refines our understanding of myoclonus in neurodegenerative disorders. It provides objective, real-time insights into the complex interplay of brain and muscle activity, guiding accurate diagnosis, informing prognosis, and ultimately contributing to more targeted and effective patient care.

## Data Availability

The raw data supporting the conclusions of this article will be made available by the authors, without undue reservation.

## Supplementary material

SUPPLEMENTARY FIGURE 1 Classification of myoclonus in neurodegenerative disorders with the three main groups: dementias, basal ganglia disorders, and spinocerebellar degenerations.

SUPPLEMENTARY VIDEO 1 Video documentation of two patients (average age 81 years) with severe Alzheimer’s disease, demonstrating myoclonic jerks, predominantly observed in the upper limbs. The myoclonic jerks are focal/segmental, and can be synchronous or asynchronous, occasionally appearing generalized/massive. They occur both at rest and are action-induced/triggered by movement.

SUPPLEMENTARY VIDEO 2 Video-EEG/polygraphic documentation of two patients with progressive myoclonic epilepsy in down syndrome (DS), with an average age of 45, presenting with myoclonic jerks. These jerks affect all four limbs, occur both at rest and are triggered by movement and posture. They can be synchronous or asynchronous and show a corresponding EEG correlate of diffuse P-PPO abnormalities. Additionally, photic stimulation at medium frequencies elicits a diffuse photo-myoclonic response in Patient 1 and a focal response in the right upper limb in Patient 2.

SUPPLEMENTARY VIDEO 3 Video-EEG/polygraphic documentation of a 36-year-old patient with Familial Alzheimer’s Disease (PSEN1 gene mutation). This video illustrates multifocal, parcellar, and massive, synchronous and asynchronous myoclonic jerks observed in all four limbs during periods of quiet wakefulness and fluctuating vigilance. The myoclonic jerks could be precipitated by movement and were associated with a vermian and hemispheric cerebellar syndrome, as well as apraxia, aphasia, and agnosia. These jerks were not consistently associated with epileptiform abnormalities.

SUPPLEMENTARY VIDEO 4 Video and video-EEG/polygraphic documentations of a patient with Creutzfeldt-Jakob disease. This video displays rhythmic, subcontinuous myoclonus in all four limbs, which was exacerbated by movement and showed a predominance in the right upper limb. These jerks frequently coincided with periodic generalized epileptiform discharges on EEG. While tactile stimulation and movement accentuated the myoclonus, there was no response to auditory stimuli.

SUPPLEMENTARY VIDEO 5 Patient with corticobasal degeneration presenting with focal, reflex myoclonus in the left upper limb, elicited by tactile stimuli. This myoclonus is notably superimposed on a distal tremor also

SUPPLEMENTARY VIDEO 6 Patient with progressive myoclonic ataxia presenting with parcellar or massive myoclonic jerks involving the trunk and upper limbs, precipitated by movement and posture.
