# Supplementary figures and images for: Navigating the diagnostic challenges of myoclonus in neurodegenerative disorders: video-EEG/polygraphy, clinical vignettes, and narrative analysis
Source: Front Neurol. 2025 Sep 12;16:1655455. doi: 10.3389/fneur.2025.1655455 (PMC12463611; doi:10.3389/fneur.2025.1655455)

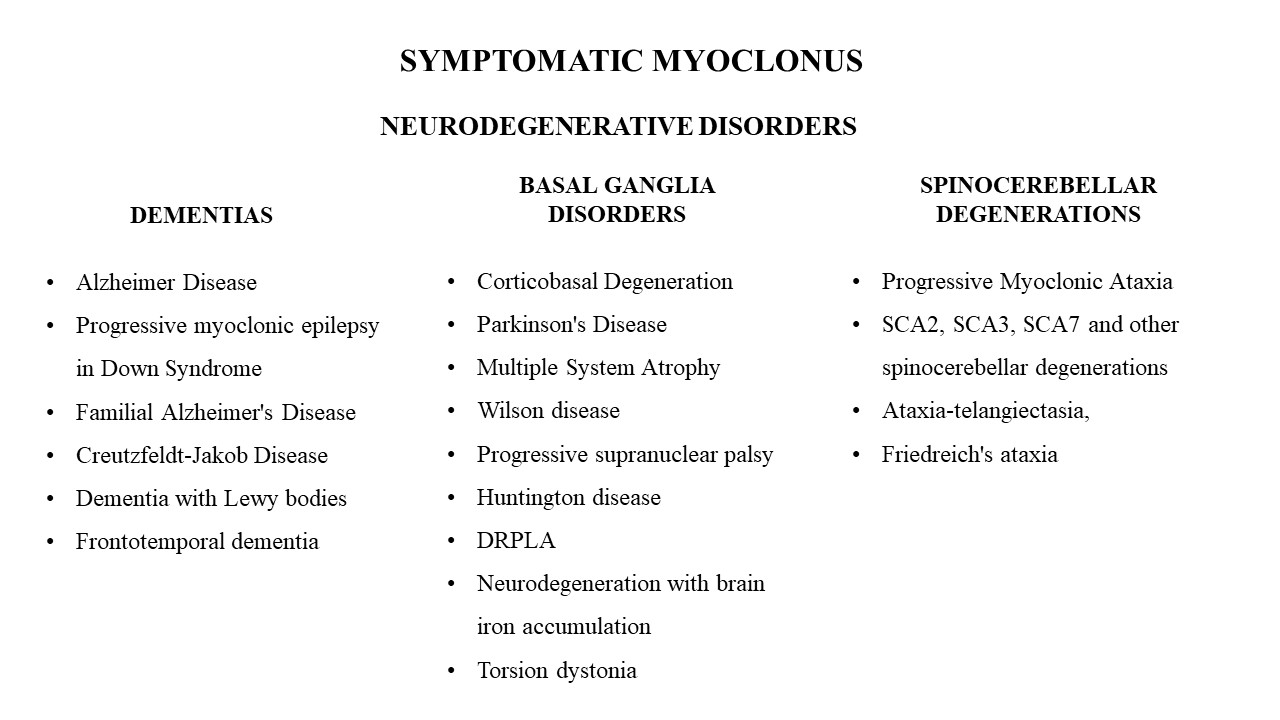

Supplement: SUPPLEMENTARY FIGURE 1 — Classification of myoclonus in neurodegenerative disorders with the three main groups: dementias, basal ganglia disorders, and spinocerebellar degenerations. [file Image_1.JPEG]
